# Detection of *BRAF* V600E in Fine-Needle Aspiration Samples of Thyroid Nodules by Droplet Digital PCR

**DOI:** 10.1155/2022/6243696

**Published:** 2022-03-29

**Authors:** Sang-Yu Lu, Ying-Chao Chen, Jia-Lin Feng, Qin-Yi Zhou, Jing Chen, Chen-Fang Zhu, Miao-Miao Guo, Man-Man Zhang, Qian-Yue Zhang, Meng Lu, Liu Yang, Jing Wu, Shuang-Xia Zhao, Huai-Dong Song, Xiao-Ping Ye

**Affiliations:** ^1^Department of Molecular Diagnostics, the Core Laboratory, Medical Center of Clinical Research, Department of Endocrinology, Shanghai Ninth People's Hospital, State Key Laboratory of Medical Genomics, Shanghai Jiao Tong University School of Medicine, Shanghai, China; ^2^Institute and Department of Endocrinology and Metabolism, Shanghai Ninth People's Hospital, Shanghai Jiao Tong University School of Medicine, Shanghai, China; ^3^Head and Neck Surgery, Ren Ji Hospital, Shanghai Jiao Tong University School of Medicine, Shanghai, China; ^4^Department of General Surgery, Shanghai Ninth People's Hospital, Shanghai Jiao Tong University School of Medicine, Discipline Construction Research Center of China Hospital Development Institute, Shanghai Jiao Tong University, Shanghai, China

## Abstract

**Background:**

*BRAF* exon 15 p.V600E (*BRAF* V600E) mutation has been established as an important molecular marker for papillary thyroid carcinoma diagnosis by ultrasound-guided fine-needle aspiration biopsy (FNAB). Sanger sequencing is the gold standard for detecting *BRAF* V600E mutations but fails to identify low-frequency mutations. However, droplet digital PCR (ddPCR) is a popular new method for detecting low-frequency mutations. Here, we compare the efficiency of droplet digital PCR (ddPCR) and Sanger sequencing for detection of the *BRAF* V600E mutation in thyroid fine-needle aspiration (FNA) samples.

**Methods:**

Thyroid fine-needle aspiration samples from 278 patients with 310 thyroid nodules were collected. Sanger sequencing and ddPCR were conducted to detect the *BRAF* V600E mutation.

**Results:**

The *BRAF* V600E mutation was found in 94 nodules (30.32%) by ddPCR and 40 nodules (12.90%) by Sanger sequencing in 310 FNA samples. A total of 119 nodules were confirmed PTC by postsurgical pathology. Among which the *BRAF* mutation was found in 80 (67.23%) nodules by ddPCR and 31 (26.05%) by Sanger sequencing. All nodules carrying the mutation detected by Sanger sequencing (SS+) were verified by ddPCR (ddPCR+). Also, all nodules with no mutation detected by ddPCR were interpreted as wild-type by Sanger sequencing (SS−). In addition. Almost all SS+/ddPCR + nodules (95.00%; 38/40) and SS−/ddPCR + nodules (100.00%; 54/54) displayed a *BRAF* mutation rate of >5% and <15%, respectively, indicating easy misdetection by Sanger sequencing when the mutation rate is between 5 and 15%.

**Conclusion:**

ddPCR has higher sensitivity than Sanger sequencing and we propose ddPCR as a supplement to Sanger sequencing in molecular testing of *BRAF* using FNAB samples.

## 1. Introduction

Thyroid carcinoma is the most common endocrine malignancy with the fastest growing incidence, among which papillary thyroid carcinoma (PTC) accounts for the vast majority (90%) [1–4]. Ultrasound-guided fine-needle aspiration biopsy (FNAB) is the most accurate preoperative test for diagnosis of PTC, significantly improving the detection sensitivity of malignant thyroid nodules [5, 6]. However, up to one-third of nodules remain cytologically undetermined and are diagnostic challenges for endocrinologists and pathologists [7, 8]. In recent decades, the limitation of FNAB cytology in PTC diagnosis has been overcome by molecular analysis using validated genetic alterations; for example, *BRAF* exon 15 p.V600E (*BRAF* V600E) mutation is an important molecular marker for PTC diagnosis with a mutation frequency of 45–60% [9–12]. The *BRAF* V600E mutation is highly specific and associated with more aggressive clinical and pathological PTC features [12–15].

Multiple strategies with various sensitivities including Sanger sequencing, allele-specific amplification PCR (ASA-PCR), amplification-refractory mutation system (ARMS-PCR), and others have been widely used to detect *BRAF* V600E mutations in FNAB [16–19]. Among these methods, Sanger sequencing is the simplest and the gold standard for mutation detection requiring a 7%–20% mutant fraction [2, 20, 21]. However, there may be few mutant cancer cells in FNA samples, which results in indeterminate or nondiagnostic cytology. Thus, a refined detection method with higher sensitivity is needed.

Droplet digital PCR (ddPCR) is a promising technique with superior sensitivity, enabling the detection and accurate measurement of trace nucleic acids. The limit of detection on *BRAF* V600E by ddPCR is reported to be only 0.0005%, which renders it an optimal method to detect the mutation in thyroid FNA samples [22]. Previous studies have reported the superior sensitivity of ddPCR over Sanger sequencing and ARMS-PCR though the cohort was small [23].

In this study, we compared the sensitivity of ddPCR and Sanger sequencing in detecting *BRAF* V600E in a large group of thyroid FNA samples. ddPCR showed a better sensitivity than Sanger sequencing. However, given the high cost of ddPCR, we recommend applying a reasonable combination of Sanger sequencing and ddPCR for clinical detection of the *BRAF* V600E mutation.

### 1.1. Patients and Methods

#### 1.1.1. Patients and Samples of FNAB Thyroid Tissue

In total, 278 patients with 310 thyroid nodules (30 patients with 2 nodules; 1 patient with 3 nodules) were enrolled in the study at the Department of Endocrinology, Shanghai Ninth People's Hospital, from May 2020 to August 2021. All patients provided informed consent, and the study was approved by the Ethics Committee of Shanghai Ninth People's Hospital (CRC/IRB-C-BD-16-V3.1, ethics no. SH9H-2020-T346-1). US-guided FNAB of all nodules was performed under a standardized protocol by an experienced endocrinologist. Material from the needle passing through the nodule was used to prepare a direct smear for cytological evaluation, and the remaining material plus the needle washing was used for molecular testing. The collection of material for molecular testing was conducted to ensure routine cytological evaluation. Cytological diagnosis was performed via cytological examination of H&E-stained FNA smears according to the diagnosis criteria of the 2017 Bethesda System for Reporting Thyroid Cytopathology [24]. A total of 191 nodules from 191 patients underwent thyroid surgery after FNAB examination, including those identified as PTC or carrying the *BRAF* V600E mutation or eligible for surgery (i.e., symptoms of oppression) or voluntarily requested due to US detection of suspicious malignancy. The surgically separated thyroid tissues were confirmed by postsurgical pathology.

### 1.2. DNA Extraction

DNA was isolated using a QIAamp DNA Micro Kit for FNAB samples (Qiagen, Germany) according to the manufacturer's protocol. Briefly, FNAB samples were collected by centrifugation and then lysed. The DNA in the lysate binds to the membrane of the QIAamp MinElute column and then eluted from the membrane after washing the membrane. The quantity of isolated DNA was assessed using a NanoDrop 8000 spectrophotometer (Thermo Scientific. USA).

### 1.3. *BRAF* V600E Mutation Detection by Sanger Sequencing

Part of exon 15 of the *BRAF* gene in which the T1799A transversion mutation (encoding *BRAF* V600E) is located was amplified by nested PCR (Supplement Table 1). The purified PCR products were sequenced using the forward primer for the second nested PCR cycle and a BigDye Terminator v 3.1 kit (Thermo Fisher, USA). Data analysis and interpretation were performed with SeqMan Pro 7.1.0 (DNASTAR) by visual inspection.

### 1.4. *BRAF* V600E Mutation Detection by ddPCR

ddPCR was performed with the QX200 Droplet Digital PCR system (cat. 1863026; Bio-Rad Technologies, USA) per the manufacturer's protocol to confirm the *BRAF* V600E mutation. Amplification was performed as follows: 95°C for 10 minutes (1 cycle), 94°C for 30 seconds, 55°C for 1 minute (40 cycles), and 98°C for 10 minutes (1 cycle) with a ramp rate of 1°C/s; the reaction was then held at 4°C with a ramp rate of 1°C/s. Quantification of mutant and wild‐type alleles was estimated using QuantaSoft v1.7.4 analysis software (Bio‐Rad Technologies, USA). The threshold was defined as described in “Droplet Digital Application Guide.” Firstly, we tested DNA from 30 FNAB and their blood samples. Of which their nodules were determined as benign by surgical pathology. Except for 1 or 2 positive events detected in 2 FNAB samples, there were no positive events in all samples. Therefore, we supposed that the sample was interpreted as *BRAF* V600E-positive when the number of positive events exceeded 3. Because a small quantity of DNA extracted from FNA samples, the total number of events of *BRAF* V600E site is less than 5000. As a result, we found all ddPCR-positive nodules shown mutation rate of >0.2%. Therefore, we proposed that the detection sensitivity of ddPCR was above 2/1000. The ddPCR-negative nodules with a total number of events less than 1000 should be excluded since insufficient events detected in each nodule may lead to false negative results. For each test, samples of benign lesions harboring no mutation and ddH_2_O were prepared as negative controls. The fractional abundance calculated and provided by the software reflects the allele frequency of the *BRAF* V600E mutation.

### 1.5. Statistical Analysis

SPSS software version 22.0 (IBM Corporation, Armonk, NY, USA) was used for statistical analysis. The *t*-test was used to compare mean values and *p* values < 0.05 were considered statistically significant.

## 2. Results

### 2.1. Cytological Identification of FNA Specimens and Pathological Identification of Surgery Specimens

Altogether, 310 nodules from 278 patients were collected: 117 nodules were diagnosed as PTC. 15 were suspicious for PTC. 13 were follicular adenoma. 1 were medullary carcinoma. 161 were benign and 3 were nondiagnostic by FNAB cytology (Table 1). A total of 191 nodules underwent surgically removed, of which 106 nodules were diagnosed as PTC by FNAB cytology, 11 as suspicious for PTC, 12 as follicular adenoma, 59 as benign lesions, and 2 as nondiagnostic nodules (Table 2). Of the 191 resected nodules, 119 nodules were diagnosed as PTC by surgery pathology, 12 as follicular adenoma, 1 as medullary carcinoma, and 59 as benign nodules.

### 2.2. ddPCR vs. Sanger Sequencing of *BRAF* V600E

**Figure 1:**
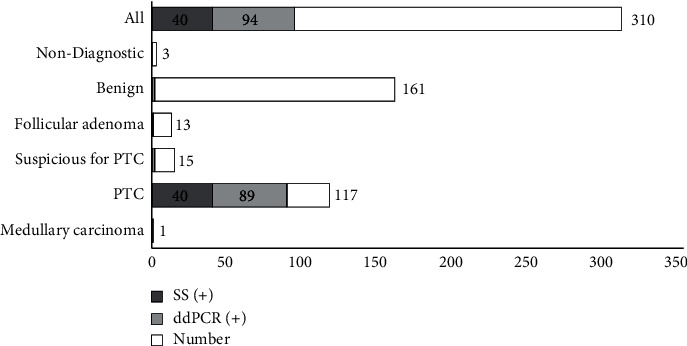
Comparison of the results of Sanger sequencing and ddPCR for *BRAF* V600E in 310 nodules of different cytological categories.

Of all 310 nodules, the *BRAF* V600E mutation was found in 94 (30.32%) by ddPCR and in 40 (12.90%) by Sanger sequencing, indicating that ddPCR was able to identify many more nodules with the *BRAF* mutation (Table 1 and Figure 1). Among 117 nodules cytologically determined as PTC, the *BRAF* V600E mutation was detected in 89 (76.07%) by ddPCR and 40 (34.19%) by Sanger sequencing. There were 5 nodules that belong to other cytological categories, including 2 nodules suspicious for PTC, 1 follicular adenoma, and 2 benign nodules, carrying *BRAF* V600E mutation detected by ddPCR but not by Sanger sequencing. All these 5 nodules displayed very low fractional abundance of the mutant allele (<2.00%), which may be explained by the small number of thyroid cancerous cells obtained by FNA (Table 3). We could not confirm the results without histological diagnosis because these 3 patients did not accept surgery due to nonmalignant FNAB. These results showed that ddPCR has higher sensitivity than Sanger sequencing in detecting *BRAF* V600E in FNA samples from nodules of different cytological categories.

**Figure 2:**
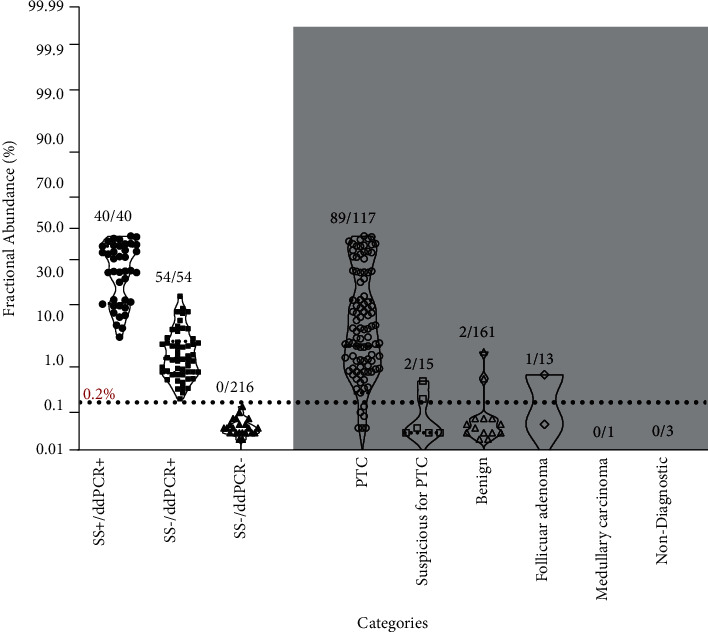
Fractional abundance of different mutation examination categories (left, white part) and FNA cytological type categories (right, gray part).

To compare the results of *BRAF* mutation detected by Sanger sequencing and ddPCR, we classified 310 nodules into three groups: Sanger sequencing-positive and ddPCR-positive group (SS+/ddPCR+), Sanger sequencing-negative and ddPCR-positive group (SS−/ddPCR+), and Sanger sequencing-negative and ddPCR-negative group (SS−/ddPCR−). None of the nodules were Sanger sequencing-positive and ddPCR-negative. The ddPCR results for nodules in the SS+/ddPCR + group displayed significantly higher fractional abundance of the mutant allele (25.05 ± 2.16 vs. 2.47 ± 0.35, *t* test: *P* < 0.0001) and more mutant events (811.38 ± 108.41 vs. 30.35 ± 6.46*. t* test: *P* < 0.0001) of *BRAF* V600E than those in the SS−/ddPCR+group (Figure 2 left; Supplement Figure 1, left). The fractional abundances of the mutant allele of all ddPCR+ nodules were >0.20%, whereas those of all ddPCR− nodules were <0.20%. Almost all SS+/ddPCR+ nodules (95.00%; 38/40) displayed a *BRAF* mutation rate >5% and all SS−/ddPCR+ nodules (100.00%; 54/54) displayed <15%, which corresponds with the detection limit of Sanger sequencing reported (Figure 2, left). These findings indicate that a mutation rate between 5 and 15% can be easily misjudged by Sanger sequencing. By the way, most PTC nodules showed more than 0.20% fractional abundance and the fractional abundance of the *BRAF* mutant allele in PTC (12.71 ± 1.54%) is higher than that in the other cytological categories (suspicious for PTC nodules: 0.37 ± 0.16%, benign nodules: 1.24 ± 0.66%, follicular adenoma: 0.07) (Figure 2, right; Table 4). This makes a lot of sense. Since FNAB can be determined as PTC by cytology, the content of cancer cells in these FNAB are definitely high and if the cancer cells are mutant, the mutation fractional abundance must be high.

**Figure 3:**
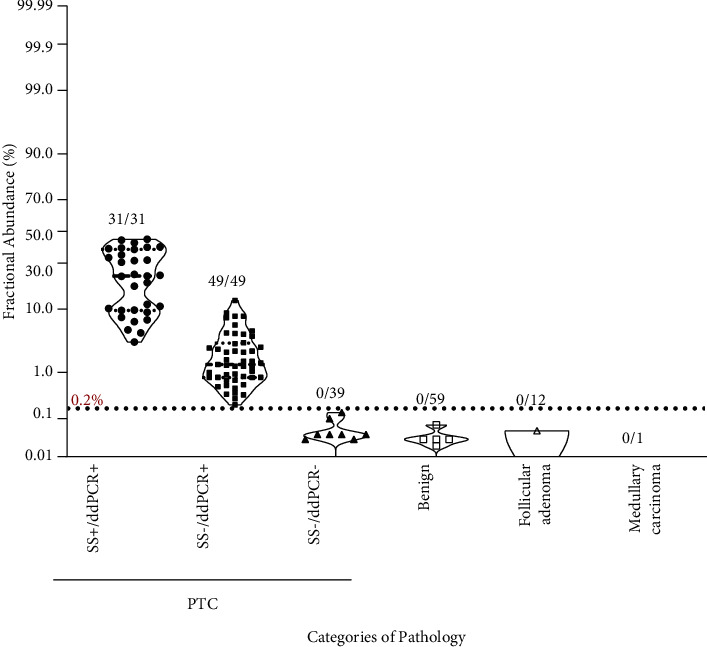
Fractional abundance of different pathological categories.

A total of 191 nodules underwent thyroid surgery after FNAB examination, of which 119 nodules were determined as PTC by postsurgical pathology. Among the 119 surgically confirmed PTC nodules, the *BRAF* mutation was found in 80 (67.23%) FANB samples by ddPCR and 31 (26.05%) by Sanger sequencing (Table 2; Figure 3; Supplement Figure 2). Eleven of 15 nodules were diagnosed as suspicious for malignancy underwent surgery, and 10 were determined as PTC by postsurgical pathology; 2 nodules were found SS−/ddPCR+ and 8 SS−/ddPCR−, whereas 1 nodule without *BRAF* mutation was diagnosed as benign lesion (Table 2). None of non-PTC nodules was found having the *BRAF* mutation by Sanger sequencing or ddPCR. The PTC nodules in the SS+/ddPCR+ group displayed higher fractional abundance of the mutant allele and having more mutant events than those in the SS−/ddPCR+ group as well (Figure 3, left; Supplement Figure 2, left). These results confirmed that ddPCR has higher sensitivity than Sanger sequencing in detecting the *BRAF* V600E mutation by using FNA samples.

### 2.3. The Value of ddPCR Application for Nodules with Indeterminate Sanger Sequencing Results

**Figure 4:**
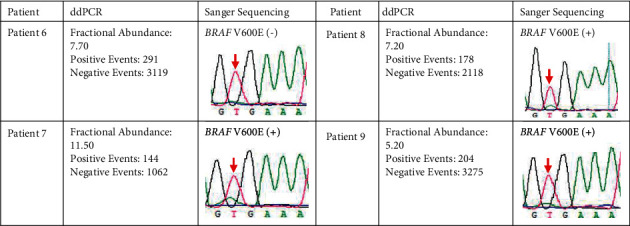
ddPCR and Sanger sequencing results for 4 patients whose Sanger sequencing results were ambiguous. Each patient in this figure had only one nodule. The *BRAF* V600E mutation is shown as red arrowheads in the Sanger sequencing chromatograms.

The above results indicate that ddPCR is a good supplement to Sanger sequencing for detection of *BRAF* V600E. There are 54 ddPCR+ nodules that were finally interpreted as negative by Sanger sequencing, and these nodules usually exhibit low mutant allele frequencies ranging from 0.2% to 15% (Figure 2, left; Figure 3, left). In addition, the SS+/ddPCR+ nodules displayed mutant allele frequencies above 5%, suggesting that nodules with 5% to 15% mutant allele frequencies could not definitely be identified by Sanger sequencing because of their ambiguous mutant peaks. ddPCR could function as further confirmation under these circumstances. For example, the mutant peaks of nodules from patients 6, 7, 8, and 9 shown by Sanger sequencing do not completely correspond to the mutant allele frequencies shown by ddPCR (Figure 4). All these 4 cases were determined as PTC based on histological pathology. Overall, interpretation of Sanger sequencing is subjective and highly dependent on the exact sequence and laboratory performance. Therefore, ddPCR might be applied to supplement the detection of nodules with negative Sanger sequencing, avoiding missed diagnosis or misjudgment due to uncertain Sanger sequencing results.

## 3. Discussion

In this study, we applied ddPCR to detect the *BRAF* V600E mutation in FNA samples from thyroid nodules and compared its sensitivity with that of Sanger sequencing. Our results showed that ddPCR has higher sensitivity in detecting the *BRAF* V600E mutation in FNA samples than Sanger sequencing (30.32% vs. 12.90%). As previously reported, we regard ddPCR as a powerful confirmatory test for Sanger sequencing-negative or indeterminate nodules.

In the current study, we preliminarily analyzed a large number of FNAB samples to determine a proper threshold of ddPCR for our laboratory, an important basis for *BRAF* mutation judgment. The threshold is susceptible to many factors such as the quality and quantity of the input DNA and the performance of the detection kit. For each test, a laboratory should establish its own judgment threshold. In this study, we set 3 positive events as the threshold; thus, the fractional abundances of all ddPCR+ nodules we found in the current study were >0.20%, and those of all ddPCR− nodules were <0.20%, which corresponded with 2/1000 detection sensitivity and indicated that the positive threshold we set was reasonable. There were 2 benign nodules and one follicular adenoma nodule showing the *BRAF* mutation by ddPCR with very low fractional abundance (<2.00%), which could not be confirmed by histological pathology since these three patients did not receive surgery. The discrepancy of cytology and *BRAF* mutation may be due to FNAB bias, which is common in biopsy, especially when the tumor is small. Regardless, due to the bias of FNA, the molecular testing result may not reflect the true situation, especially for those with low mutation rate (fractional abundance <0.20%) and indetermined cytology. Thus, we recommend another FNAB or regular follow-up for these patients.

**Figure 5:**
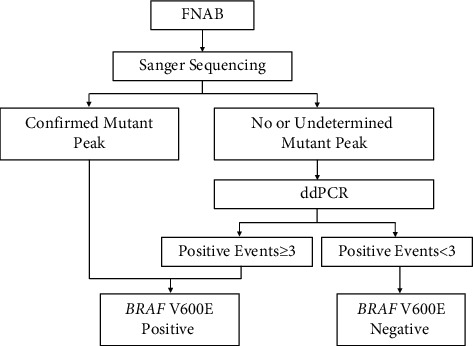
The flowchart of clinical detection of the *BRAF* V600E mutation we suggest. The positive threshold of ddPCR should be adjusted according to the different conditions of different laboratories.

Considering the high cost of ddPCR, we suggest a reasonable combination of Sanger sequencing and ddPCR for the clinical detection of the *BRAF* mutation in FNAB samples. Our results demonstrate that nodules with mutation rate of >15% can be easily and stably detected by Sanger sequencing but that nodules with mutation rate of <5% cannot; nodules with mutation rate of 5 to 15% usually have uncertain Sanger sequencing results. ARMS is another method used for clinical detection of *BRAF* mutation and has been compared with ddPCR in other studies. However, ARMS is considered not as suitable as Sanger sequencing because it has relatively low specificity and may detect benign lesions as false positive [23, 25]. Hence, we propose the pipeline shown in Figure 5 for molecular testing of *BRAF* using FNAB. Nodules with definite mutant peaks by Sanger sequencing are considered to carry the *BRAF* mutation, whereas those with uncertain or no mutant peak should be further assessed by ddPCR. The appropriate positive threshold of ddPCR should be determined by individual laboratory.

## Figures and Tables

**Table 1:** Comparison of the results of Sanger sequencing and ddPCR for *BRAF* V600E in 310 nodules with FNAB cytology results.

Bethesda categories	Cytologic reporting	N	ddPCR(+)	SS(+)
All	All	310	94	40
Bethesda I	Nondiagnostic	3	0	0
Bethesda II	Benign	161	2	0
Bethesda III	Follicular adenoma	13	1	0
Bethesda V	Suspicious for PTC	15	2	0
Bethesda VI	PTC	117	89	40
Bethesda VI	Medullary carcinoma	1	0	0

PTC, papillary thyroid carcinoma; ddPCR, droplet digital PCR; SS, sanger sequencing.

**Table 2:** Comparison of the results of Sanger sequencing and ddPCR for *BRAF* V600E in 191 nodules with pathology results.

FNAB		Surgery						
Bethesda categories	Cytologic reporting	N	PTC	ddPCR(+)	SS(+)	Non-PTC	ddPCR(+)	SS(+)
All	All	191	119	80	31	72	0	0
Bethesda I	Nondiagnostic	2	0	0	0	2	0	0
Bethesda II	Benign	59	3	0	0	56	0	0
Bethesda III	Follicular adenoma	12	0	0	0	12	0	0
Bethesda V	Suspicious for PTC	11	10	2	0	1	0	0
Bethesda VI	PTC	106	106	78	31	0	0	0
Bethesda VI	Medullary carcinoma	1	0	0	0	1	0	0

PTC, papillary thyroid carcinoma; ddPCR, droplet digital PCR; SS, sanger sequencing; non-PTC, including benign nodules, follicular adenoma, and medullary carcinoma.

**Table 3:** The *BRAF* mutation result in SS−/ddPCR+ nodules suspicious for PTC, follicular adenoma, and benign categories.

Nodule	Sanger sequencing	ddPCR			Cytologic reporting
		Fractional abundance (%)	Positive events	Negative events	
Nodule 1	Negative	0.21	5	2112	Suspicious for PTC
Nodule 2	Negative	0.52	20	3317	Suspicious for PTC
Nodule 3	Negative	0.70	3	442	Follicular adenoma
Nodule 4	Negative	0.58	16	2454	Benign
Nodule 5	Negative	1.90	30	1492	Benign

**Table 4:** Fractional abundance of the *BRAF* mutation in FNAB of different cytological categories.

Cytologic reporting	N	Fractional abundance			
		≥0.2%		＜0.2%	
		*n*	%	*n*	%
PTC	117	89	76.07	29	24.79
Suspicious for PTC	15	2	13.33	13	86.67
Benign	161	2	1.24	159	98.76
Follicular adenoma	13	1	7.69	12	92.31
Medullary carcinoma	1	0	0.00	1	100.00
Nondiagnostic	3	0	0.00	3	100.00

## Data Availability

The data that support the findings of this study are available on request from the corresponding author. The data are not publicly available due to privacy or ethical restrictions.

## Supplementary Materials

Supplementary Materials Supplement Table 1: procedure of nested PCR to amplify exon 15 of the *BRAF* gene. Supplement Figure 1: positive events of different mutations of examination categories (left, white part, and FNA cytological categories). Supplement Figure 2: positive events of different pathological categories (right, grey part).
