# Supplementary material for: Detection of BRAF V600E in Fine-Needle Aspiration Samples of Thyroid Nodules by Droplet Digital PCR
Source: Int J Endocrinol. 2022 Mar 29;2022:6243696. doi: 10.1155/2022/6243696 (PMC8983273; doi:10.1155/2022/6243696)

Supplement Table 1 Procedure of nested PCR to amplify exon 15 of the *BRAF* gene

**1st nest PCR mix**			
Component	Concentration	Volume (ul)	
DNA from FNA sample	10-100 ng/µl	20-100 ng (1-2 µl)	
Taq DNA Polymerase	-	0.5	
UNG ase	-	0.5	
Buffer	10X	2	
Q Solution	5X	4	
dNTP (dUTP)	2-4 mM	1	
BRAF FP-1*	10 mM	0.5	
BRAF RP-1*	10 mM	0.5	
H_2O	-	Up to 20	
Total		20	
**1st nest PCR amplification procedure**			
Step	Temperature	Time	
Degrade U-DNA	50	2 min	1 cycle
Initial Denaturation	94	5 min	1 cycle
Denature	94	40 sec	35 cycles
Anneal	58	40 sec	
Extend	72	60 sec	
Final Extension	72	10 min	1 cycle
Hold	4	∞	1 cycle
**2st nest PCR mix**			
Component	Concentration	Volume (µl)	
Products from 1st 1:10 dilution	-	2	
Taq DNA Polymerase	-	0.5	
Buffer	10X	2	
Q Solution	5X	4	
dNTP (dUTP)	2-4 mM	1	
BRAF FP-2*	10 mM	0.5	
BRAF RP-2*	10 mM	0.5	
H_2O	-	Up to 20	
Total		20	
**2st nest PCR amplification procedure**			
Step	Temperature	Time	
Initial Denaturation	94	5 min	1 cycle
Denature	94	30 sec	35 cycles
Anneal	56	40 sec	
Extend	72	45 sec	
Final Extension	72	10 min	1 cycle
Hold	4	∞	1 cycle

*Primers (5' to 3'):

BRAF-FP-1: TTGCTCTGATAGGAAAATGAGATCTACT
BRAF-RP-1: TCAGTGGAAAAATAGCCTCAATTCT
BRAF-FP-2: TACTGTTTTCCTTTACTTACTACACCTCAG
BRAF-RP-2: GGAAAAATAGCCTCAATTCTTACCAT

Supplement Figure 1 Positive events of different mutation examination categories (left, white part) and FNA cytological categories (right, gray part).

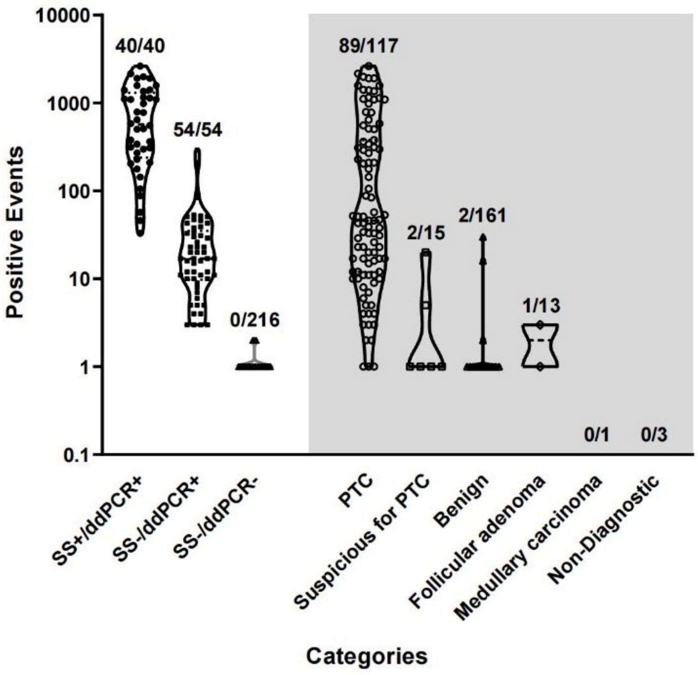

Supplement Figure 2 Positive events of different pathological categories.

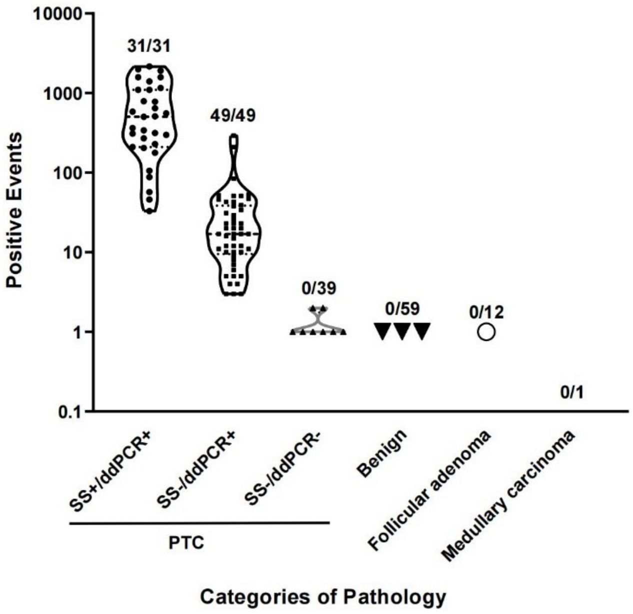

Supplement: Supplementary Materials — Supplement Table 1: procedure of nested PCR to amplify exon 15 of the BRAF gene. Supplement Figure 1: positive events of different mutations of examination categories (left, white part, and FNA cytological categories). Supplement Figure 2: positive events of different pathological categories (right, grey part). [file 6243696.f1.pdf]
